# Association between preoperative right heart catheterization parameters and outcomes in patients undergoing isolated coronary artery bypass grafting

**DOI:** 10.1093/icvts/ivae158

**Published:** 2024-09-13

**Authors:** Irbaz Hameed, Ralf Martz Sulague, Eric S Li, Doruk Yalcintepe, Katherine Candelario, Andrea Amabile, Victory B Effiom, Haleigh Larson, Arnar Geirsson, Matthew L Williams

**Affiliations:** Division of Cardiac Surgery, Yale University School of Medicine, New Haven, CT, USA; Division of Cardiac Surgery, Yale University School of Medicine, New Haven, CT, USA; Division of Cardiac Surgery, Yale University School of Medicine, New Haven, CT, USA; Division of Cardiac Surgery, Yale University School of Medicine, New Haven, CT, USA; Division of Cardiac Surgery, Yale University School of Medicine, New Haven, CT, USA; Division of Cardiac Surgery, Yale University School of Medicine, New Haven, CT, USA; Division of Cardiac Surgery, Yale University School of Medicine, New Haven, CT, USA; Division of Cardiac Surgery, Yale University School of Medicine, New Haven, CT, USA; Division of Cardiac Surgery, Yale University School of Medicine, New Haven, CT, USA; Division of Cardiac Surgery, Yale University School of Medicine, New Haven, CT, USA

**Keywords:** CABG, PCI, coronary heart disease

## Abstract

Right ventricular catheterization may capture information that can help define prognosis before coronary artery bypass grafting (CABG). In this study, we evaluate the association between preoperative right heart catheterization parameters and outcomes of patients undergoing isolated CABG. All patients undergoing isolated CABG at our institution from 2013 to 2021 who also underwent preoperative right heart catheterization <14 days prior to isolated CABG were retrospectively queried. A total of 2343 patients underwent isolated CABG of whom 78 patients [20 (25.6%) female] were included in the final analysis. On multivariable regression, central venous pressure was significantly associated with operative mortality (odds ratio 1.14, 95% confidence interval 1.02–1.27, *P* = 0.024). Preoperative cardiac index was significantly inversely associated with intensive care unit length of stay (odds ratio 0.72, 95% confidence interval 0.62–0.84, *P* < 0.001) and duration of inotropic support (odds ratio 0.76, 95% confidence interval 0.63–0.92, *P* < 0.01). Assessment of preoperative cardiac function by right heart catheterization should be considered in high-risk patient populations, particularly those who have significant left ventricular dysfunction on preoperative echocardiography that would make them candidate for percutaneous coronary intervention, left ventricular assist device or heart transplantation. Further, right heart catheterization can help to guide preoperative optimization and intra-/postoperative decision-making.

## Graphical Abstract

**Figure:**
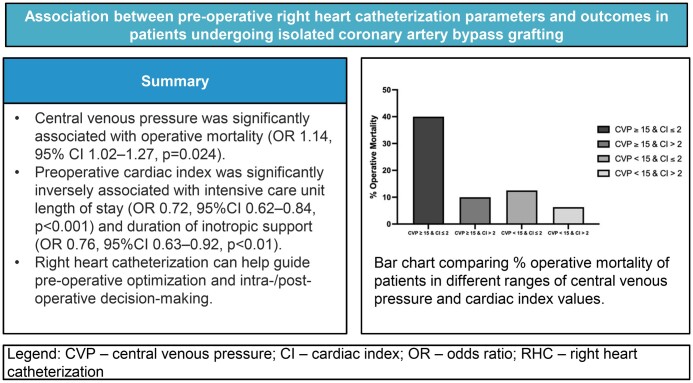

## INTRODUCTION

Cardiac dysfunction is a major cause of morbidity and mortality in adult cardiac surgery. Right ventricular catheterization may capture information that can help define prognosis before coronary artery bypass grafting (CABG). Variables such as pulmonary artery, systolic and diastolic pressures, overall cardiac output (indexed to patient size) and central venous pressure (CVP) may all be correlated to outcome after CABG and may be useful in determining therapy when percutaneous coronary intervention, durable mechanical circulatory support or heart transplant are under consideration in patients with very poor left ventricular function and coronary artery disease.

The right ventricle exhibits a particularly greater sensitivity to changes in afterload. This can foster a vicious cycle in which the right ventricle dilates, leading to a shift in the septum, a modification in the left ventricular geometry and eventually a reduction in the left ventricular contractility [1]. This eventually leads to right ventricular dilation and dysfunction, which manifests clinically as hypotension and cardiogenic shock.

In CABG, while several clinical and left ventricular variables have been shown to be associated with patient prognosis and postoperative outcomes [2], there are limited data on the impact of preoperative right heart catheterization on patient outcomes and prognosis. The majority of previous studies on the right heart have focused on the impact of CABG on postoperative right heart dysfunction [3].

We aimed at evaluating the association between preoperative right heart catheterization parameters and outcomes of patients undergoing isolated CABG.

## METHODS

All patients undergoing isolated CABG at our institution from 2013 to 2021 who also underwent preoperative right heart catheterization <14 days prior to isolated CABG were retrospectively queried. The indications for right heart catheterization included patients presenting with shock where the aetiology was unclear or when precise haemodynamic measurements were required for management, patients with signs of pulmonary hypertension and patients with heart failure and severely diminished left ventricular function on preoperative echocardiography. Patients with coronary artery disease are evaluated by a multidisciplinary team of general and interventional cardiologists and cardiac surgeons to determine the choice of treatment including medical therapy, percutaneous intervention or CABG. Patients with heart failure are additionally evaluated by advanced heart failure cardiology teams and closely followed postoperatively and after discharge.

The following parameters were recorded for each patient: CVP, right ventricular pressure, pulmonary artery pressure, pulmonary capillary wedge pressure, cardiac index and cardiac output. Patient comorbidities were obtained from the Join Data Analytics Team database.

The primary outcome was operative mortality. Secondary outcomes were postoperative myocardial infarction, stroke, need for dialysis, duration of intensive care unit (ICU) stay and duration of inotropic support. Operative mortality was defined according to the definitions of the Society of Thoracic Surgeons Adult Cardiac Surgery Database [4]. Myocardial infarction (MI) was defined according to the Fourth Universal Definition of Myocardial Infarction [5]. Stroke was defined as central nervous system infarction based on neuropathological, neuroimaging and/or clinical evidence of neurologic dysfunction [6]. The study protocol conforms to the ethical guidelines of the 1975 Declaration of Helsinki as reflected in a priori approval by the Yale University Human Investigation Committee.

Continuous variables were recorded as mean (standard deviation) or median (interquartile range) following assessment of normality using Shapiro–Wilk test for normality. Categorical variables were recorded as counts and percentages. Multivariable regression adjusting for the Society of Thoracic Surgeons predicted risk of mortality score was performed to evaluate association of variables with postoperative outcome. Multicollinearity was assessed using the variance inflation factor. Results were reported as the exponent of the regression coefficient [Exp (β)] and its 95% Wald confidence interval (CI).

## RESULTS

A total of 2343 patients underwent isolated CABG of whom 78 patients [20 (25.6%) female] were included in the final analysis. The median age [interquartile range] was 67 [60, 74]. Fifty-three (67.9%) patients had prior MI, 28 (35.9%) had a prior stroke and 38 (48.7%) had renal dysfunction. Forty-four (56.4%) patients had peripheral vascular disease, and 44 (56.4%) had diabetes. The mean (standard deviation) preoperative left ventricular ejection fraction was 33.3% (±14.4). Patient characteristics are summarized in Table 1.

**Table 1:** Patient demographics, preoperative characteristics and postoperative outcomes

	RHC with CABG (*n* = 78)
Characteristics	
Age (years)	67 [60, 74]
Female	20 (25.6%)
Myocardial infarction	53 (67.9%)
Renal dysfunction	38 (48.7%)
Hypertension	63 (80.8%)
Hyperlipidaemia	59 (75.6%)
Diabetes	44 (56.4%)
Stroke	28 (35.9%)
Peripheral vascular disease	44 (56.4%)
Outcomes	
Operative mortality	8 (10.3%)
Stroke	5 (6.4%)
Myocardial infarction	7 (9.0%)
Need for dialysis	4 (5.1%)
Major adverse events (operative mortality + stroke + myocardial infarction + need for dialysis)	16 (20.5%)
Duration of ICU stay (days)	4 [3.0, 7.8]
Duration of inotropic support (days)	2 [1.3, 4.0]

Values are *n* (%) or median [IQR] with units of measure as shown.

CABG: coronary artery bypass grafting; ICU: intensive care unit; RHC: right heart catheterization.

The incidence of operative mortality was 8 (10.3%). The incidence of the secondary outcomes were: 7 (9.0%) for MI, 5 (6.4%) for stroke and 4 (5.1%) for need of dialysis. The median durations of ICU stay and inotropic support in days were 4 (3.0, 7.8) and 2 (1.3, 4.0), respectively. Sixteen (20.5%) experienced any major adverse event (operative mortality, MI, stroke or need for dialysis). Details of postoperative outcomes are summarized in Table 1.

On multivariable regression, CVP was significantly associated with operative mortality [odds ratio (OR) 1.14, 95% CI 1.02–1.27, *P* = 0.024]. Preoperative cardiac index was significantly inversely associated with ICU length of stay (OR 0.72, 95% CI 0.62–0.84, *P* < 0.001) and duration of inotropic support (OR 0.76, 95% CI 0.63–0.92, *P* < 0.01).

**Figure 1:**
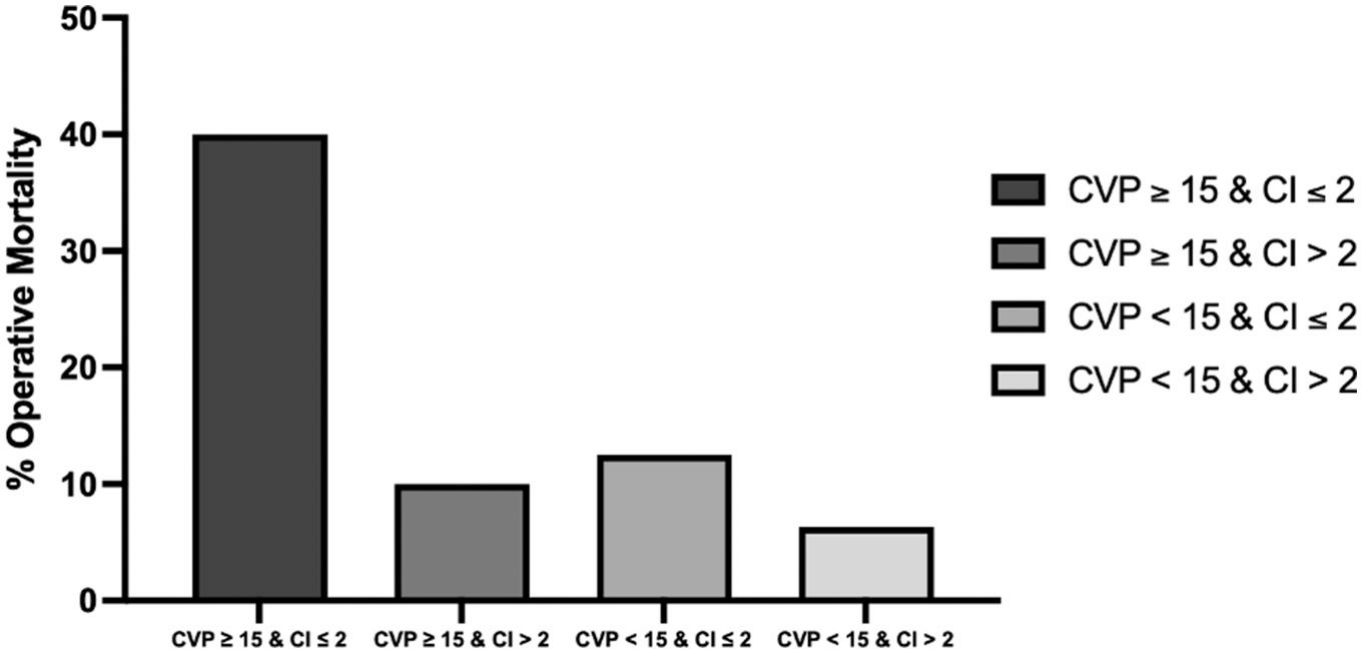
Bar chart comparing % operative mortality of patients in different ranges of central venous pressure (CVP) and cardiac index (CI) values.

On stratifying patients by CVP and CI values, the highest incidence of operative mortality (*n* = 4, 40%) was observed in patients with CVP ≥ 15 and CI ≤ 2. Conversely, the lowest incidence of operative mortality (*n* = 2, 6.3%) are observed in patients with CVP < 15 and CI > 2. Patients with CVP ≥ 15 and CI > 2 and those with CVP < 15 and CI ≤ 2 had an operative mortality incidence of 10% (*n* = 1) and 12.5% (*n* = 1), respectively (Fig. 1).

## DISCUSSION

Our study evaluated the association between preoperative right heart catheterization parameters and postoperative outcomes in patients undergoing isolated CABG. After adjusting for baseline risk factors, we found high preoperative CVP to be independently associated with higher operative mortality and prolonged postoperative inotropic support. Similarly, low preoperative cardiac index was significantly associated with prolonged ICU stay and inotrope support.

Current literature on preoperative right heart dysfunction and association with postoperative outcomes in patients undergoing cardiac surgery is sparse. Previous data on right heart catheterization are limited to postoperative catheterization parameters.

In a 2014 study of 2390 patients undergoing isolated CABG, Williams *et al.* found CVP measured 6 h postoperatively to be significantly associated with higher 30-day mortality (OR 1.5, 95% CI 1.23–1.87 for every 5-mmHg increase in CVP, *P* < 0.0001). This association remained significant after adjustment for cardiac index (adjusted OR 1.44, 95% CI 1.10–1.89), *P* < 0.01) [7]. Their study, however, was limited to patients with left ventricular ejection fraction <40% or age ≥65 with either diabetes mellitus or a glomerular filtration rate <60 ml/min.

In another study of 200 patients undergoing CABG, Sumin *et al*. [8] assessed preoperative right ventricular diastolic dysfunction by transthoracic echocardiography and evaluated its association with CABG outcomes. They found preoperative right ventricular diastolic dysfunction to be significantly associated with post-operative heart failure [OR 3.39 (95% CI 1.15–9.97; *P* = 0.025]. Their observations were not based on right heart catheterization parameters but subjective echocardiographic assessments and institutional definition of right heart dysfunction. In this context, the applicability of their findings to other institutions and clinical scenarios is difficult to ascertain. Additionally, while echocardiography is renown to be an operator-dependent diagnostic technique, right heart catheterization produces less nuanced findings.

We found elevated preoperative CVP and low pre-operative cardiac index to be significantly associated with worse prognosis despite adjustment for STS-PROM. While cut-offs for defining right heart dysfunction are not clear, current data shows post-operative CVP > 20 mmHg and a CI < 2.1 l/min/m to be associated with worse prognosis [9]. Ahmed *et al.* [10] in a previous study showed a CI ≤2.5 l/min/m^2^ to be predictive of inotrope need following concomitant CABG and aortic valve replacement surgery. This is consistent with our observation.

Low cardiac output following isolated CABG is associated with higher mortality, morbidity and prolonged ICU stay. One of the underlying mechanisms of postoperative low cardiac output syndrome includes right ventricular dysfunction. Preoperative right heart catheterization can allow better stratification of patients with cardiogenic shock who are high risk for surgery. These patients may benefit from preoperative volume optimization by diuresis and other interventions, improvement of right ventricular contractility by restoring sinus rhythm and inotropic support, and decreasing afterload if pressure overload is deemed contributory. Percutaneous coronary intervention or durable mechanical circulatory support or heart transplant may also be considered in patients meeting eligibility criteria. The highest incidence of operative mortality in our series was noted in patients with a preoperative CVP ≥15 mmHg and CI ≤2 l/min/m^2^; for patients who met both of these criteria, operative mortality was notably nearly 40%. These patients should be considered for preoperative hospital admission, central line placement, CVP-guided aggressive diuresis and medical optimization.

### Limitations

Our study shares the limitations of all retrospective observational studies. Our sample size was small, and larger future studies are certainly required to further detail the prognostic value of right heart catheterization in this setting. Our study was also limited to a single-centre, albeit high-volume, and our findings may not be generalizable to other institutions. Right heart parameters are dynamic, and it was not possible to record them at different preoperative intervals. Our study, however, is the 1st to provide objective data on prognostic associations of preoperative right heart catheterization parameters. These must be interpreted in the context of patients’ other baseline comorbidities during pre-operative evaluation.

## CONCLUSION

In conclusion, despite the inclusion of cardiogenic shock in STS-PROM, baseline CVP and cardiac index are independently associated with operative mortality, inotrope requirement, and duration of ICU stay in patients undergoing isolated CABG. Assessment of preoperative cardiac function by right heart catheterization should be considered in high-risk patient populations, particularly those who have significant left ventricular dysfunction on preoperative echocardiography that would make them candidate for percutaneous coronary intervention, left ventricular assist device or heart transplantation. Further, right heart catheterization can help to guide preoperative optimization and intra-/postoperative decision-making.

## Data Availability

The data that support the findings of this study are available from the corresponding author, Dr Irbaz Hameed, upon reasonable request. The data are not publicly available due to restrictions from the Yale University Human Investigation Committee, which ensure participant privacy and confidentiality.

## Notes

Presented at the American Heart Association Scientific Session 2022, Chicago, Illinois, 5–7 November 2022.
